# Adaptive Test Schemes for Control of Paratuberculosis in Dairy Cows

**DOI:** 10.1371/journal.pone.0167219

**Published:** 2016-12-01

**Authors:** Carsten Kirkeby, Kaare Græsbøll, Søren Saxmose Nielsen, Lasse Engbo Christiansen, Nils Toft, Tariq Halasa

**Affiliations:** 1 DTU VET, Section for Epidemiology, Technical University of Denmark, Denmark; 2 Department of Large Animal Sciences, Section for Animal Welfare and Disease Control, University of Copenhagen, Denmark; 3 DTU Compute, Section for Dynamical Systems, Department of Applied Mathematics and Computer Science, Technical University of Denmark, Richard Petersens Plads, Denmark; University of Minnesota, UNITED STATES

## Abstract

Paratuberculosis is a chronic infection that in dairy cattle causes reduced milk yield, weight loss, and ultimately fatal diarrhea. Subclinical animals can excrete bacteria (*Mycobacterium avium* ssp. *paratuberculosis*, MAP) in feces and infect other animals. Farmers identify the infectious animals through a variety of test-strategies, but are challenged by the lack of perfect tests. Frequent testing increases the sensitivity but the costs of testing are a cause of concern for farmers. Here, we used a herd simulation model using milk ELISA tests to evaluate the epidemiological and economic consequences of continuously adapting the sampling interval in response to the estimated true prevalence in the herd. The key results were that the true prevalence was greatly affected by the hygiene level and to some extent by the test-frequency. Furthermore, the choice of prevalence that will be tolerated in a control scenario had a major impact on the true prevalence in the normal hygiene setting, but less so when the hygiene was poor. The net revenue is not greatly affected by the test-strategy, because of the general variation in net revenues between farms. An exception to this is the low hygiene herd, where frequent testing results in lower revenue. When we look at the probability of eradication, then it is correlated with the testing frequency and the target prevalence during the control phase. The probability of eradication is low in the low hygiene herd, and a test-and-cull strategy should probably not be the primary strategy in this herd. Based on this study we suggest that, in order to control MAP, the standard Danish dairy farm should use an adaptive strategy where a short sampling interval of three months is used when the estimated true prevalence is above 1%, and otherwise use a long sampling interval of one year.

## Introduction

Paratuberculosis is a chronic infection caused by *Mycobacterium avium* ssp. *paratuberculosis* (MAP). Most infected dairy cattle are believed to be infected as calves from MAP shed in the feces by infectious animals, and subsequently transmitted via the environment and feed for oral uptake [1]. MAP can be shed by subclinical as well as clinical cattle, and while the progress of infection can be slow, the subclinical phase can last for several years [2]. Subclinical dairy cows are subject to lower milk yield and lower slaughter value, causing an economic loss for the dairy farmer [3,4,5]. Ultimately, the clinical phase can result in diarrhea and death. Timely culling can therefore prevent not only clinical disease, but also spread of the infection to susceptible herd mates.

Many countries have voluntary or mandatory test schemes for paratuberculosis in order to control MAP nationally (e.g. Australia: [6]), or within single herds (e.g. Denmark: [7]), or to prove freedom of infection nationally (Sweden: [8]) or within single herds (Denmark: [9]). Detection of infectious animals can be done using MAP specific antibody ELISA (for serum or milk), or bacteriological culture or PCR on fecal samples [10].

A variety of tests and test strategies exist, and not all tests are fit for all purposes [10]. Furthermore, the balance between test-and-cull, and management only needs to be considered under the conditions prevailing [11]. In Denmark, the ID Screen ELISA test (IDvet, Grabels, France) is used in the voluntary national control program for paratuberculosis. Herd screens of lactating cows are done quarterly and cows are classified based on up to the four latest ELISA results [12]. This classification basically results in the separation of cows into three risk categories, high, medium and low risk cows, used to manage the cows on a day-to-day basis to reduce transmission, particularly around calving [12]. The costs for testing are a concern for farmers and can prevent them from entering, or discourage their continuous participation in a voluntary control program, when the prevalence in a herd has dropped to a low level. When the prevalence is low, subclinical animals are rarely identified and clinical animals might not occur at all. The farmer may therefore think that the costs for testing are wasted, even though testing actually helps to keep the prevalence at a low level on the farm, and the net revenue is not noticeably affected (see [13] for the economic impact of paratuberculosis). We here propose a novel test-scheme with potential for reducing costs, while still maintaining the control of paratuberculosis in a herd. This new test-scheme adapts according to the prevalence in the herd and uses a long sampling interval when the within-herd prevalence is low, and a shorter one only when required, thus saving costs for testing.

Simulation models are useful to explore the potential of different strategies within a farm [14], [15]. This is especially true regarding paratuberculosis because it is slowly developing and lack of perfect tests. The aim of this study was to evaluate the epidemiological and economic effects of an adaptive test strategy where farmers can adjust the sampling intervals according to the prevalence in their herd. This was done through a simulation study using a bio-economic model, where the effect of a flexible sampling interval, and an adaptive strategy on the epidemiological and economic effects was evaluated in a dairy cow herd with high and low prevalence

## Materials and Methods

We used the iCull model which is a mechanistic, stochastic and dynamic model that simulates individual cows in a standard Danish dairy cattle herd with daily time steps [13]. All simulations comprised a herd with 200 dairy cows and were run for 10 years, which followed a 3 year burn-in period, using 500 repetitions. The model simulates individual milk ELISA test strategies which are currently used in the Danish paratuberculosis control program [12]. In the model, the milk yield of the individual cow is adjusted according to their infection progress following [16], so that the higher the ELISA value, the lower the milk yield (further described in [13]). The within-herd prevalence can be very different between farms [17]. Therefore we simulated all testing scenarios both in a herd with an initial true prevalence of 5.6% (corresponding to a herd with a median prevalence and thus “normal” level of hygiene [13]) and in a herd with a high prevalence (with an initial prevalence of 45% corresponding to a “low” level of hygiene [13]). We simulated a closed herd (i.e. without introduction of livestock) in all simulations, which is common in 50% of all dairy herds in Denmark [13].

All simulated scenarios used the test-and-cull strategy, which has previously been shown to be sufficient for reducing the prevalence [13], [14], [18]. Test-and-cull is based on repeated ELISA test results for each cow so that repeated test-positive cows are marked for culling [12]. Baseline scenarios used a non-adaptive sampling interval fixed at three months. This test interval is used in all Danish herds participating in the paratuberculosis control program. The adaptive test-schemes in this study operate with two test intervals; short sampling intervals (abbreviated SSI, testing often to lower the prevalence) and long sampling intervals (abbreviated LSI, testing less often). In order to have comparable scenarios, all scenarios started were initiated with a sampling interval of 3 months during the burn-in period. In all simulations, the farmer used a test-and-cull strategy where repeated test-positive cows were marked for culling [13]. In the simulations, the true prevalence was continuously estimated based on the latest test results from the herd (i.e. the apparent prevalence), using the Rogan-Gladen estimator [19]:

$$TP\;=\;\frac{\text{AP }+\text{ Sp }-\;1}{\text{Sp }+\text{ Se }-\;1}$$

where TP = true prevalence, AP = apparent prevalence, Sp = test specificity (98.66%), Se = test sensitivity (age dependent, see [13]). We simulated scenarios with three specified tolerance levels for the prevalence, namely 1%, 3% and 5%, reflecting the temperament of the farmer. We name this tolerance level, or prevalence cutoff PC. The 1% level simulates farmers that are highly concerned about paratuberculosis and want to eradicate or keep the prevalence at a very low level. The 3% and 5% levels simulated farmers that are less concerned about paratuberculosis and just wants to control the disease, i.e. an endemic situation. When the estimated true within-herd prevalence exceeded the chosen tolerance level, the farmer started to test with the short sampling interval (SSI, Table 1). When the estimated true prevalence dropped below the tolerance level, the farmer tested with the long sampling intervals (LSI). The simulated test-schemes are listed in Table 1.

**Table 1:** The parameters used in the study. In the simulations where the initial prevalence was 5.6. The prevalence cutoff is the level of within-herd prevalence that the farmer tolerates in a control scenario.

Parameters	Setting	Unit
Long sampling interval, LSI	365 / 730	Days
Short sampling interval, SSI	31 / 91 / 182	Days
Initial prevalence	5.6 / 45	%
Prevalence cutoff	1 / 3 / 5	%

The economics in the simulations are summarized over the full 10 year simulation period as described in detail in [13]. Briefly, costs are recorded for feed, insemination, rendered animals and ELISA tests. The simulated specificity of the ELISA test is 98.66% and the sensitivity is dependent on the progression of disease, increasing up to 79%[20]. The income is generated from milk sold at a fixed (default) price, slaughtered animals and sold bull calves. Animals infected with MAP have a reduced slaughter value according to [5]. The estimated net revenue was thus the income minus the costs. This model does neither include the general labor costs or costs for housing, machines etc. Likewise, the costs for testing do not include labor because this task can be performed by the farmer when milking.

During the simulations we saved data on the number of simulated days before a farm switched to LSI (meaning that the prevalence tolerance level was reached). We also recorded if the farmer switched back to SSI, because the prevalence again exceeded the prevalence tolerance level. Using these data, we calculated the probability of switching to LSI and the probability of switching back to SSI once LSI was reached. We also saved data on the true prevalence and calculated the probability of eradicating MAP from the herd.

## Results

**Fig 1:**
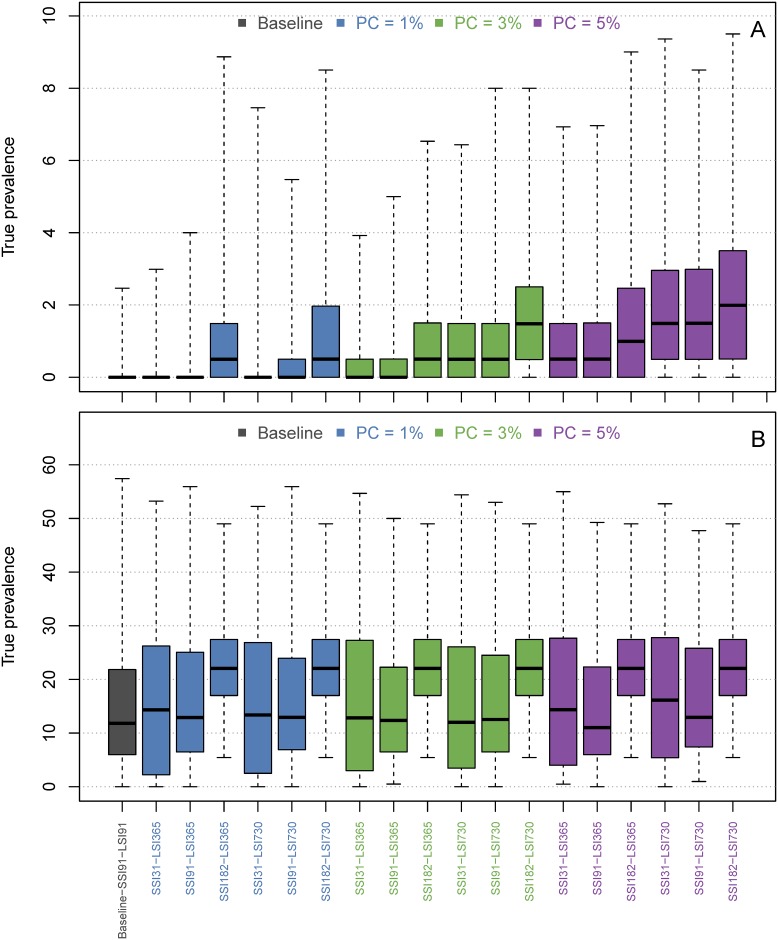
Boxplots of the true prevalence distributions after ten simulated years for each scenario for the normal hygiene herd (A) and the low hygiene herd (B). In these scenarios the farmer chose a prevalence cutoff (PC) as the maximum MAP prevalence they will tolerate. When the prevalence exceeds this value, a short sampling interval is used. When the prevalence is lower than this value, a long sampling interval is used. X-axis labels show the short sampling interval and long sampling interval. SSI = Short sampling interval, LSI = Long sampling interval. F. ex., SSI31-LSI365 means that SSI is 31 days and the LSI is 365 days. The solid bar is the median value, the box represents 25th–75th percentiles and whiskers show range of values. In the baseline scenario, the test interval is fixed to every three months. Therefore there is almost no variation in the costs for testing. This scenario results in a mean prevalence around 12% in the low-hygiene herd and zero in the normal hygiene herd. The total mean annual net revenue on the farm did not differ much between the simulated scenarios, except from when the SSI was set to monthly testing where the net revenue decreased noticeably (Fig 2). When looking at the costs for ELISA testing separately (normal hygiene herd, PC = 1%), the median annual costs in the normal hygiene herd varied between 1300 EUR and 4000 EUR (Fig 3), i.e. between 6.5 and 20 EUR per cow-year. These costs became even lower when the farmer chose a higher PC. In the baseline scenario, about 2750 EUR was spent annually on ELISA testing. In the low hygiene herd (PC = 1%), the ELISA costs varied from 1400 EUR to 8000 EUR (Fig 3). The costs did not vary when using different values of PC. The baseline costs for ELISA testing in the low hygiene herd were the same as in the normal hygiene herd.

We generally found that the higher the tolerance level is in the adaptive test scheme, the higher the prevalence will be (Fig 1A). Furthermore, the lower the SSI and LSI, the faster the prevalence was reduced (Fig 1A). For the low hygiene herd, the prevalence was generally reduced to medians in the range 10%-15%, except when SSI was 182 days, in which the resulting median prevalences were in the range 20%-25% (Fig 1B).

**Fig 2:**
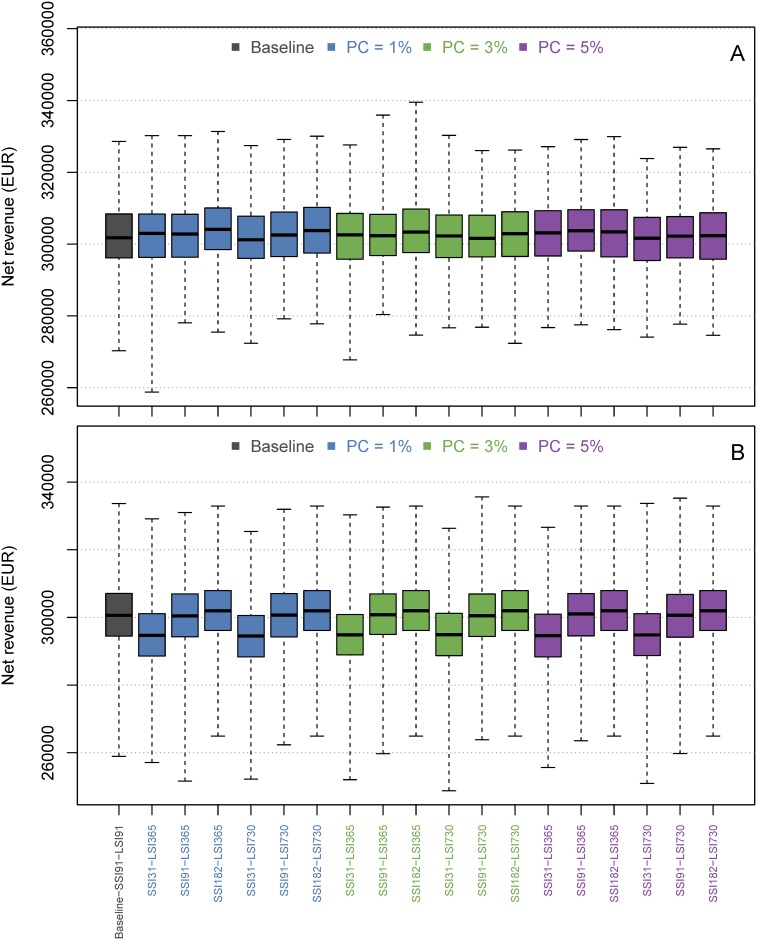
Boxplots of the mean annual net revenue (EUR, over ten simulated years) on the farm of ten simulated years for each scenario for the normal hygiene herd (A) and the low hygiene herd (B). X-axis labels show the short sampling interval (SSI), long sampling interval (LSI) and prevalence cutoff (PC).

**Fig 3:**
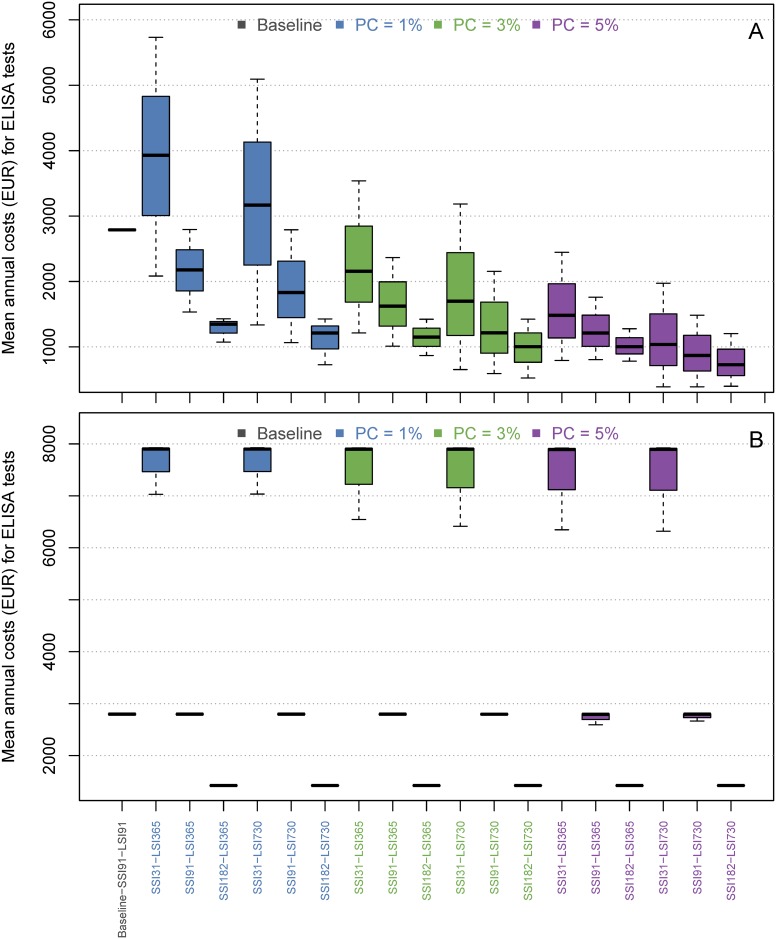
Boxplots of the distribution of mean annual costs (EUR, over ten simulated years) for ELISA testing for each scenario for the normal hygiene herd (A) and the low hygiene herd (B). X-axis labels show the short sampling interval (SSI), long sampling interval (LSI) and prevalence cutoff (PC).

The probability of eradication in the normal hygiene herd was highest when SSI/LSI was 31/365 (Table 2). In the low hygiene herd, the probability of eradication was generally very low between 0% and 7% (Table 3).

**Table 2:** Summary statistics for the simulated scenarios in the normal hygiene herd, showing the median number of days passed before switching to LSF with 90% simulation envelope (5%-95%), the probability of switching to LSI and the probability of switching back to SSI given that LSI was reached. PC = Prevalence cutoff, SSI = Short sampling interval, LSI = Long sampling interval. The baseline scenario is currently used in Denmark with a fixed three-monthly test interval.

	Scenario	Days before LSI			Prob. of eradication	Prob. of LSI	Prob. of SSI
		Median	5%	95%			
	Baseline	**-**	**-**	-	0.87		
PC = 1%	SSI31-LSI365	849	212	1824	0.92	1	0.94
	SSI91—LSI365	1640	361	3259	0.78	0.89	0.82
	SSI182-LSI365	1911	452	3364	0.43	0.64	0.8
	SSI31-LSI730	863	209	1886	0.79	1	0.72
	SSI91-LSI730	1726	361	3182	0.69	0.9	0.5
	SSI182-LSI730	1927	452	3364	0.39	0.71	0.48
PC = 3%	SSI31-LSI365	418	119	863	0.72	1	0.91
	SSI91-LSI365	940	179	2272	0.61	1	0.83
	SSI182-LSI365	1273	88	3000	0.4	0.92	0.79
	SSI31-LSI730	438	117	956	0.48	1	0.64
	SSI91-LSI730	913	179	2013	0.43	1	0.52
	SSI182-LSI730	1377	143	3127	0.25	0.89	0.48
PC = 5%	SSI31-LSI365	257	88	553	0.4	1	0.79
	SSI91-LSI365	520	88	1362	0.41	1	0.75
	SSI182-LSI365	756	88	2090	0.34	1	0.7
	SSI31-LSI730	248	88	491	0.24	1	0.55
	SSI91-LSI730	516	88	1271	0.24	1	0.46
	SSI182-LSI730	717	88	1908	0.17	0.99	0.5

**Table 3:** Descriptive statistics for the simulated scenarios in the low hygiene herd, showing the median number of days passed before switching to LSI with 90% simulation envelope (5%-95%), the probability of switching to LSI and the probability of switching back to SSI given that LSI was reached. SSI = Short sampling interval, LSI = Long sampling interval. The baseline scenario is currently used in Denmark with a fixed three-monthly test interval.

	Scenario	Days before LSI			Prob. of eradication	Prob. of LSI	Prob. of SSI
		Median	5%	95%			
	Baseline	**-**	**-**	**-**	0	**-**	**-**
PC = 1%	SSI31-LSI365	3089	2340	3591	0.07	0.15	0.43
	SSI91-LSI365	3136	2454	3523	0.01	0.01	0.5
	SSI182-LSI365	-	-	-	0	0	-
	SSI31-LSI730	3182	2478	3622	0.04	0.11	0.15
	SSI91-LSI730	-	-	-	0	0	-
	SSI182-LSI730	-	-	-	0	0	-
PC = 3%	SSI31-LSI365	3024	2312	3591	0.03	0.19	0.46
	SSI91-LSI365	3350	2995	3637	0	0.04	0.25
	SSI182-LSI365	-	-	-	0	0	-
	SSI31-LSI730	3096	2188	3617	0.03	0.21	0.21
	SSI91-LSI730	3302	2818	3637	0	0.05	0.16
	SSI182-LSI730	-	-	-	0	0	-
PC = 5%	SSI31-LSI365	3013	2131	3591	0	0.29	0.52
	SSI91-LSI365	3230	2650	3637	0	0.13	0.34
	SSI182-LSI365	-	-	-	0	0	-
	SSI31-LSI730	3010	2146	3591	0	0.26	0.25
	SSI91-LSI730	3234	2472	3637	0	0.07	0.14
	SSI182-LSI730	-	-	-	0	0	-

In the low hygiene scenarios, a large LSI of two years generally did not increase the prevalence much because the LSI was rarely used. This was because the prevalence was too high to trigger a switch to LSI. However, in the normal hygiene scenarios a LSI of 730 days resulted in a higher prevalence compared to a LSI of 365 days, most pronounced when PC = 3 and 5 (Fig 1).

For the normal hygiene herd with PC = 1%, the simulation with the lowest median number of days passed before switching to LSI, was 849 days in the scenario with SSI/LSI: 31/365 (Table 2). For the low hygiene herd, the scenario with the lowest median number of days passed before switching to LSI (PC = 1%), was after 3089 days with SSI/LSI: 31/365. The probability of switching to LSI was high for the normal hygiene herd, decreasing when increasing the SSI (Table 2). For the low hygiene herd, the probability of switching to LSI was generally low, and it was further decreasing with increasing SSI (Table 3). The probability of switching back to SSI once LSI was reached, was also fairly high in all scenarios, with an inverse correlation to the PC used. The probability of switching back to SSI when LSI was reached was above 50% in most normal hygiene scenarios (Table 2).

**Fig 4:**
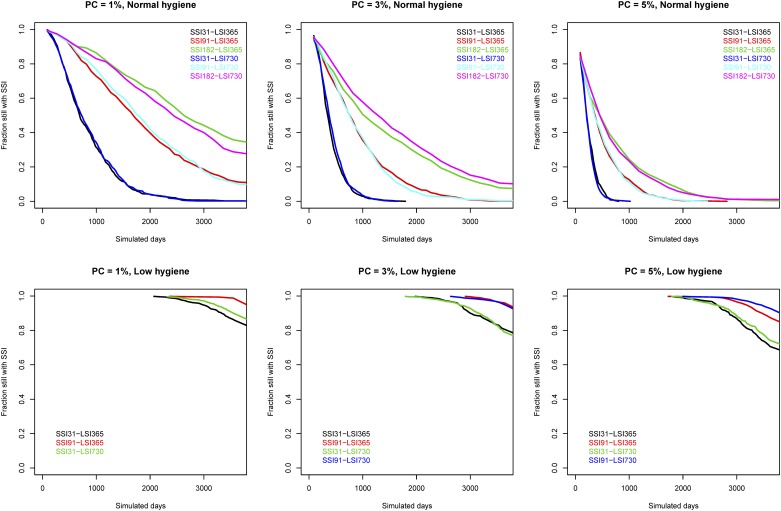
Kaplan-Meier plots showing the fraction of simulations that reached LSI over time. PC indicated the prevalence cutoff used.

We plotted the fraction of simulations that reached LSI over time in Fig 4. The shown Kaplan-Meier plots are right censored so that if some simulations did not reach LSI, the plot line will not reach zero. The fastest scenarios to reach LSI is when PC = 5% and SSI = 31. In the low hygiene herd, most scenarios do not reach LSI.

**Fig 5:**
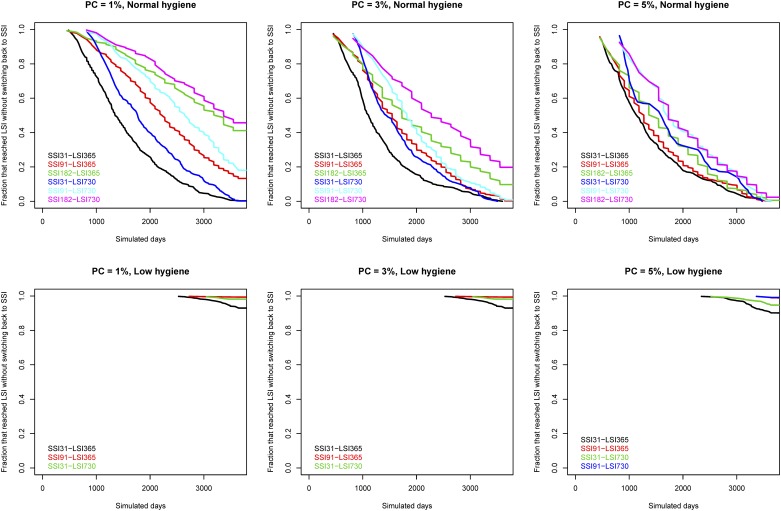
Kaplan-Meier plots showing the fraction of the simulations where LSI was reached without switching back to SSI.

In Fig 5 we show the fraction of simulations that reached LSI without switching back to SSI. In the normal hygiene herd, the scenario with SSI = 365 and LSI = 730 resulted in the highest proportion of simulations that did not switch back to SSI. In the low hygiene herd, most simulations do not reach LSI and therefore few switches back to SSI again.

## Discussion

We here proposed a novel adaptive test-scheme for controlling paratuberculosis within a herd. The results generally showed that the adaptive test-scheme can be used for saving costs while still reducing the prevalence, depending on the starting prevalence and the simulated strategy. Compared with the baseline scenario in the normal hygiene herd (PC = 1%), we found that using a SSI of 91 days and LSI of 365 did not increase the median prevalence, but decreased the median annual costs for ELISA testing from 2750 (baseline) to 2250 EUR in a herd of 200 cows (Fig 3A).

In the low hygiene herd, costs for ELISA testing was not reduced in scenarios with SSI = 91 and LSI = 365, and it did not change the prevalence in the herd (PC = 1%, Fig 3B).

A long SSI of 182 days generally resulted in the highest prevalence (Fig 1) and took longest time to reach LSI (Fig 4). Decreasing the SSI to the current baseline interval of 91 days caused the prevalence to drop the most, a feature most distinct in the low hygiene scenarios (Fig 1B). This also decreased the time before LSI was reached (Fig 4). Decreasing the SSI further (to 31 days) caused the simulations to reach LSI earlier, but caused the interquartile range to increase (Fig 1). This effect is likely due to the short SSI, which is useful for eradicating test-positive animals in the short run, but the overall test-sensitivity and ability to detect subclinical animals does not increase. This means that subclinical animals can still be present in the herd when the farmer switches to LSI, allowing MAP infections to progress further without timely detection. Thus, it is not recommended to have a very short SSI when using an adaptive test strategy, because it can actually increase the prevalence.

A large LSI of 730 days was not useful for reducing the prevalence (Fig 1). This means that farmers who want to control the disease, should avoid using LSI of 730, because this interval allows the prevalence to increase between tests.

The model does not calculate the costs for managing the adaptive sampling strategy (e.g. in a web-interface). However, if an adaptive test system is to be used, it should preferably be switching automatically between LSI and SSI and tell the farmer which sampling interval is used. The model also does not account for the labor costs for sampling, so in the scenarios where less frequent testing is used, the economic benefit may be even higher. Furthermore, we here only took into account the costs for testing with milk ELISA, as currently used in the Danish milk testing scheme, but the sampling costs will be much higher if serum is used.

In Table 2 we see that using a higher tolerance level, PC, in the normal hygiene herd generally increases the probability of reaching LSI (meaning that the prevalence is below the tolerance level, PC). Once LSI was reached, scenarios with higher PC had less risk of switching back to SSI. In the low hygiene scenarios, the probability of switching back to SSI was much less, but this is most likely because the prevalence started high in these scenarios so less time was simulated with a low prevalence (Table 3). However, we interpret these results so the adaptive approach should be a permanent solution rather than a temporary means of reducing the prevalence. This is most likely caused by the more aggressive transmission pattern in the low hygiene herd where the probability of infection from the environment is higher than in the normal hygiene herd [13].

The sampling interval has been subject to discussion for many years. [21] speculated that testing once a year was probably sufficient for a successful test-and-cull program. As stated in[22], monthly testing during lactation will increase the probability of detecting infected animals. This was also found in a study by [18] where test-and-cull was found effective with short test intervals (1 to 3 months). However, because tests are expensive in the long run, it is better to adjust the sampling interval with regards to the within-herd prevalence. From this study, we suggest to combine this approach with a higher sampling interval in times where the prevalence is higher than a given tolerance level. Mandatory or voluntary control programs for paratuberculosis are used in many countries, and recommendations for the choice of test and sampling interval are diverse [6], [7], [8], [9], [23], [24], [25]. A long sampling interval is already used in some countries, for example in the Dutch milk quality program where it is mandatory to test all lactating animals every second year. The results of our study support the usage of a long sampling interval for herds with a low prevalence. However, it is important to continuously evaluate the sampling strategy to keep the prevalence at a low level and optimize the net revenue.

Although adaptive test strategies are useful in herds with normal hygiene or a low starting prevalence, this may not be the case in the low hygiene herd. In the low hygiene herd scenarios there is limited difference in the true prevalence and the net revenue across the scenarios, but the test costs are much higher in some of the scenarios with frequent testing. This could potentially prevent farmers from testing. Simultaneously, the chance of eradication is minimal. Instead, a low hygiene (high prevalence) farm should initially focus on improving hygiene to reduce transmission as recommended elsewhere (e.g. [24]) and according to the approaches described in practice [26]. Test-and-cull strategies may be useful in some settings, but care should be exerted in just implementing these without consideration of the complexity of the infection.

## Conclusion

This is, to our knowledge, the first time an adaptive test strategy has been proposed and examined for paratuberculosis in dairy herds. Based on the results of this study, we suggest the optimal test scheme for a standard Danish farm use a short sampling interval (SSI) of three months when the true within-herd prevalence is above 1%, and a long sampling interval (LSI) of one year when the within-herd true prevalence is below 1%. This can save costs for ELISA testing while keeping the prevalence at a low level with a good probability of eradication of MAP from the herd.
